# Tabetic Arthropathy of the Knee: A Case Series of Three Patients

**DOI:** 10.7759/cureus.108639

**Published:** 2026-05-11

**Authors:** Sofia Kada, Saadia Ait Malek, Imad Ghozlani, Erraoui Mariam

**Affiliations:** 1 Rheumatology, Mohammed VI University Hospital, Agadir, MAR; 2 Rheumatology, Oued Eddahab Military Hospital, Faculty of Medicine and Pharmacy, Ibn Zohr University, Agadir, MAR; 3 Rheumatology, Mohammed VI University Hospital, Faculty of Medicine and Pharmacy, Ibn Zohr University, Agadir, MAR

**Keywords:** knee arthropathy, neurosyphilis, penicillin g, tabetic arthropathy, tertiary syphilis

## Abstract

Tabetic arthropathy is a destructive neurogenic joint disorder occurring as a late manifestation of tertiary syphilis. Although it has become exceedingly rare owing to early and appropriate treatment of syphilis, it remains relevant in developing countries and poses significant management challenges. We report three cases of tabetic arthropathy diagnosed in our department. A retrospective analysis was conducted on three cases of tabetic arthropathy diagnosed in our department between January 2022 and December 2025. Our series included three patients (n=3) with a mean age of 66 years and a male predominance (sex ratio M/F: 2/1). The main presenting complaint was chronic, painless, bilateral involvement of the knees, with a mean duration of seven months, without constitutional symptoms. The diagnosis of tabetic arthropathy was established based on painless bilateral arthropathy of the knees, characteristic radiological features combining destructive and hypertrophic osteoarticular lesions with intra-articular osteocartilaginous fragments and positive serologic tests in serum (3/3 patients), synovial fluid (3/3 patients), and cerebrospinal fluid (3/3 patients). Cardiovascular and neurological manifestations of tertiary syphilis were identified in one patient, consisting of aortic regurgitation and syphilitic cerebral arteritis. Treatment consisted of parenteral ceftriaxone due to the unavailability of penicillin G, the reference therapy. Tabetic arthropathy is a rare complication of neurosyphilis that remains relevant today in developing countries. The diagnosis should be considered in the presence of any destructive and painless joint disease. Given the difficulty in managing this articular form, prevention through early-stage treatment of syphilis is essential.

## Introduction

Tabetic arthropathy is a destructive neurogenic joint disorder. It represents a rare complication of neurosyphilis, typically developing 10-30 years after untreated or inadequately treated early syphilis [1]. With the widespread use of early and effective treatment for syphilis, neurosyphilis has become a rare, almost historical, complication. Tabetic arthropathy may present as either monoarticular or polyarticular involvement. The most frequently affected joints include the knee, followed by the ankle, tarsus, hip, dorsolumbar spine, shoulder, and elbow. Diagnosis is often delayed due to the painless nature of the condition, which is frequently disproportionate to the extent of joint destruction [2]. Imaging plays a key role in providing pathognomonic signs, the most important of which is the presence of multiple intra-articular bony formations or “loose bodies” forming a “bag of bones.” It emphasizes joint destruction and deformation [3]. In this study, we report three cases of tabetic arthropathy, aiming to better characterize their clinical, radiological, and therapeutic features. We also contextualize our findings through a review, with the objective of highlighting diagnostic challenges and optimizing the clinical management of this rare condition.

## Case presentation

Our series included three patients (n = 3) with a mean age of 66 years and a male predominance (male-to-female ratio 2:1). All patients presented with chronic, progressive, and predominantly painless bilateral knee involvement, with a mean symptom duration of seven months. Notably, no significant deterioration in general health status was observed at presentation.

We report the case of a 70-year-old man with no relevant medical history who was referred to our emergency department for advanced, painless joint destruction. Syphilis serology was reactive. His symptoms began in 2019 with the onset of inflammatory left knee pain associated with joint swelling, which responded well to nonsteroidal anti-inflammatory drugs. The clinical course was marked by recurrent, untreated flares involving both knees, ultimately leading to joint destruction and complete functional impairment. Of note, the patient reported no prior history of syphilitic chancre, roseola, or secondary syphilis.

**Figure 1:**
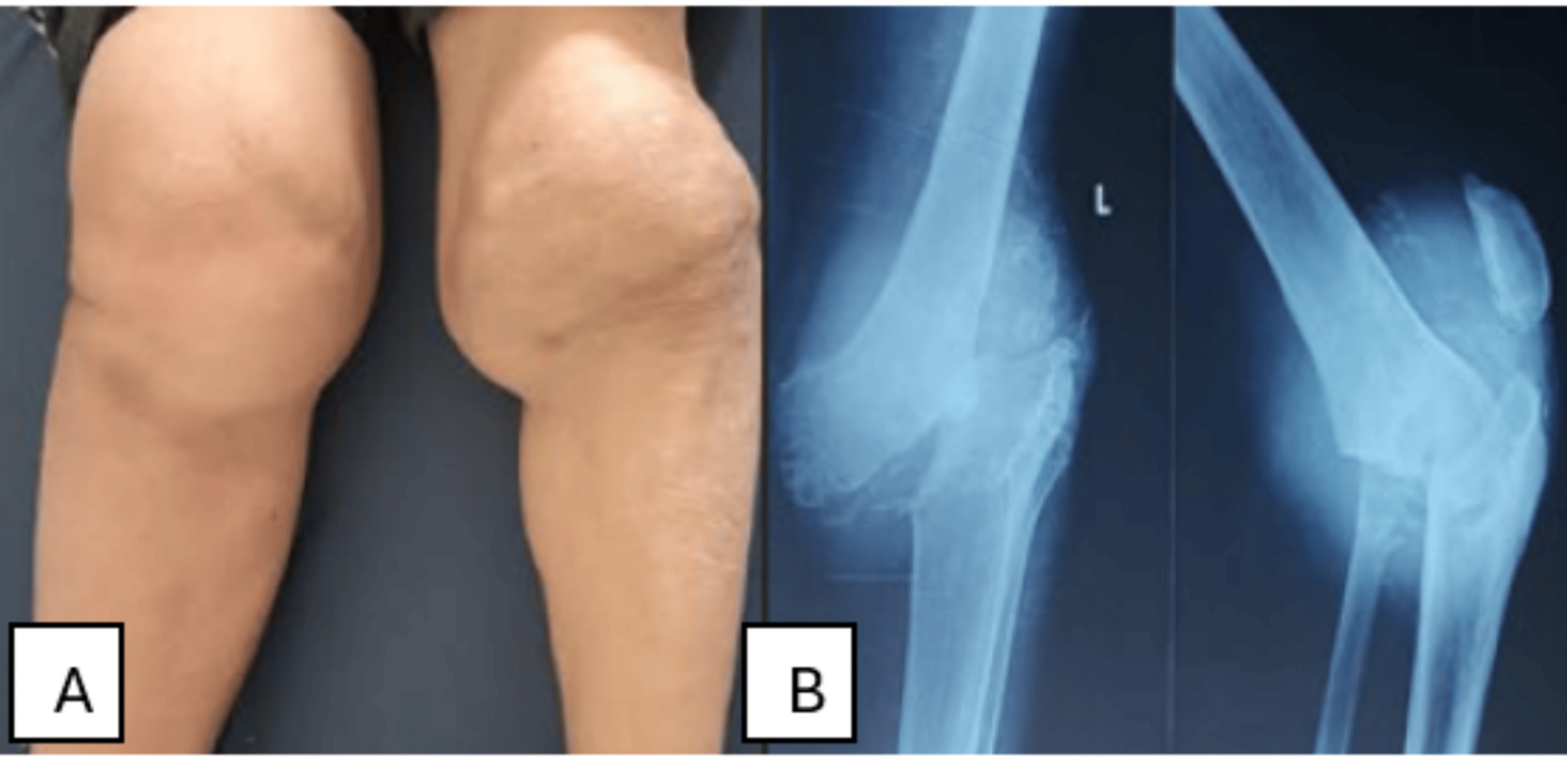
Imaging of Patient 1. (A) Clinical photograph showing the patient’s knees with structural deformation. (B) Lateral radiograph of the knees showing joint destruction and intra-articular fragments.

On musculoskeletal examination, there was marked, painless, bilateral swelling, more pronounced on the left side, with loss of normal anatomical landmarks (Figure 1A). No other osteoarticular abnormalities were noted. Neurological examination revealed impaired proprioception and vibration sense, mild lower limb hypotonia, and bilateral lower extremity areflexia. The Argyll Robertson pupil was present. Knee radiographs showed mixed destructive and sclerotic changes with intra-articular fragments and loss of joint congruity, contrasting with the absence of pain characteristic of neuropathic arthropathy (Figure 1B).

Laboratory tests demonstrated a significant inflammatory response. Treponemal and nontreponemal syphilis serologies were reactive in blood, synovial fluid, and cerebrospinal fluid (Table 1).

**Table 1:** Syphilis serology results and normal ranges (Case 1). ESR: erythrocyte sedimentation rate; CRP: C-reactive protein; CSF: cerebrospinal fluid; TPHA: treponema pallidum haemagglutination; VDRL: venereal disease research laboratory.

	Results	Reference ranges
ESR	76	<20 mm
CRP	155	<5g mg/L
Serum syphilis serology	TPHA = 1:1280 (Reactive) VDRL = 1: 32 (Reactive)	Non-reactive
Synovial fluid syphilis serology	TPHA = 1: 640 (Reactive) VDRL = 1:16 (Reactive)	Non-reactive
CSF syphilis serology	TPHA = 1:320 (Reactive) VDRL = 1:4 (Reactive)	Non-reactive

**Figure 2:**
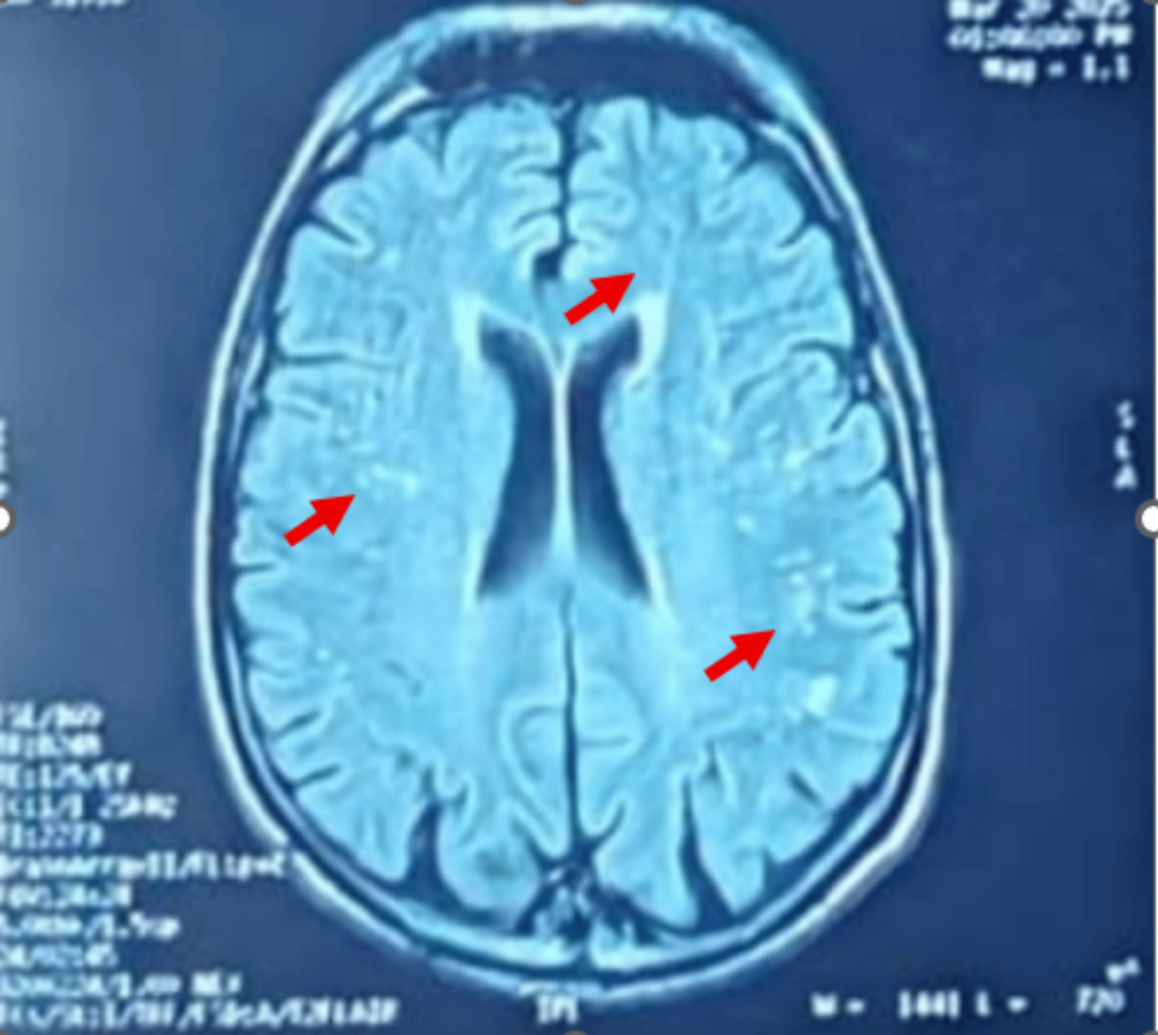
Axial FLAIR MRI sequence (Case 1). Hyperintense signal abnormalities in the periventricular and subcortical white matter, consistent with syphilitic arteritis (red arrow).

This led us to diagnose tabetic arthropathy. Further investigations were performed to assess for other manifestations of tertiary syphilis. Brain and spinal MRI revealed white matter signal abnormalities of probable vascular origin, an acute nodular ischemic stroke in the left caudate nucleus, and cortico-subcortical cerebral atrophy (Figure 2). Our neurology colleagues diagnosed syphilitic arteritis. Antiplatelet and statin therapy were initiated for secondary stroke prevention in accordance with current stroke guidelines, alongside antibiotic treatment for the underlying syphilitic arteritis.

On cardiac evaluation, a diastolic aortic murmur was noted on auscultation, and the electrocardiogram showed an incomplete left bundle branch block. Transthoracic echocardiography revealed severe aortic regurgitation and aneurysmal dilatation of the ascending aorta. Subsequent CT aortography confirmed aneurysmal dilatation of the aorta with calcified walls but without signs of complications. Our cardiology colleagues diagnosed cardiovascular syphilis. 

After consultation with the infectious disease team, the patient received a 14-day course of intravenous ceftriaxone (2 g daily) due to the unavailability of aqueous crystalline penicillin G. He was subsequently discharged with a six-month follow-up appointment. Control VDRL testing of serum, CSF, and joint fluid returned negative.

Our second case is a 72-year-old female patient who was admitted to the Neurology Department for a frontoparietal ischemic stroke presenting with right hemiparesis and Broca's aphasia. This was also attributed to syphilitic arteritis by our neurology colleagues. The patient initially presented with a progressively enlarging, painless swelling of the right knee. She had difficulty walking with an unsteady gait requiring bilateral support.

**Figure 3:**
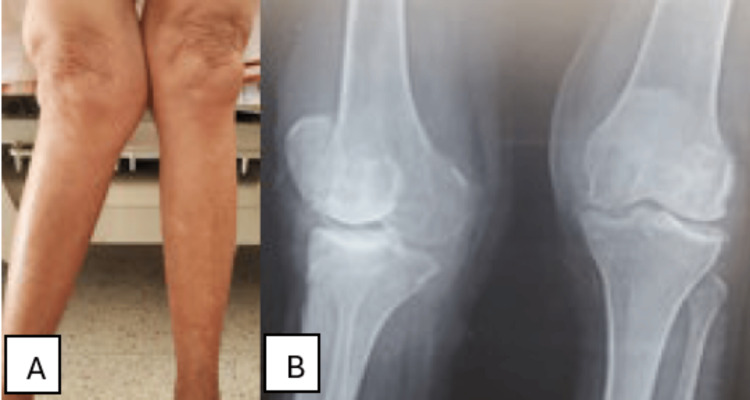
Imaging of Patient 2. (A) Clinical photograph of the knees demonstrating knee deformity. (B) Anteroposterior radiograph of both knees revealing bilateral destruction and malalignment.

On inspection, there was marked right-sided genu valgum and pelvic asymmetry (Figure 3A). On musculoskeletal examination, the right knee showed loss of normal bony landmarks with a marked patellar tap, a heel-to-buttock distance of 15 cm, and absence of pain on passive range of motion. The left knee examination revealed a painful range of motion without limitation, a positive patellar grind test, and a negative patellar tap. Neurological examination of both lower limbs demonstrated decreased muscle strength (2/5 on the right and 4/5 on the left), absent deep tendon reflexes, and diminished superficial and proprioceptive sensation. These findings, characterized by painless joint destruction in the setting of profound sensory loss and areflexia, were consistent with neuropathic arthropathy. Plain radiographs showed severe joint disorganization with rotational femorotibial subluxation and complete joint incongruence, consistent with a destructive tabetic arthropathy (Figure 3B).

Laboratory investigations revealed an inflammatory syndrome and positive syphilis serology in serum, synovial fluid, and cerebrospinal fluid (Table 2).

**Table 2:** Syphilis serology results and normal ranges (Case 2). ESR: erythrocyte sedimentation rate; CRP: C-reactive protein; CSF: cerebrospinal fluid; TPHA: treponema pallidum hemagglutination; VDRL: venereal disease research laboratory.

	Results	Reference ranges
ESR	38	<20 mm
CRP	65	<5g mg/L
Serum syphilis serology	TPHA = 1:2560 (Reactive) VDRL = 1: 64 (Reactive)	Non-reactive
Synovial fluid syphilis serology	TPHA = 1:640 (Reactive) VDRL = 1 :16 (Reactive)	Non-reactive
Cerebrospinal fluid syphilis serology	TPHA = 1:320 (Reactive) VDRL = 1:8 (Reactive)	Non-reactive

On cardiac evaluation, a holosystolic murmur was auscultated at the mitral area, and the electrocardiogram demonstrated left ventricular hypertrophy. Transthoracic echocardiography revealed mitral regurgitation with moderate left ventricular hypertrophy, which our cardiology colleagues attributed to cardiovascular syphilis.

Following consultation with the infectious disease team, and due to the unavailability of aqueous crystalline penicillin G, the patient received a 14-day course of intravenous ceftriaxone (2 g daily). She was discharged with a scheduled six-month follow-up appointment for repeat VDRL testing; however, she was unfortunately lost to follow-up.

Our third case involved a male patient referred to our outpatient clinic for bilateral knee deformities. His symptoms dated back five years, with an insidious onset of painless bilateral knee swelling and progressive varus/valgus deformity. The clinical course was marked by worsening joint instability and gait impairment despite the absence of pain. No history of trauma or primary/secondary syphilis was reported.

**Figure 4:**
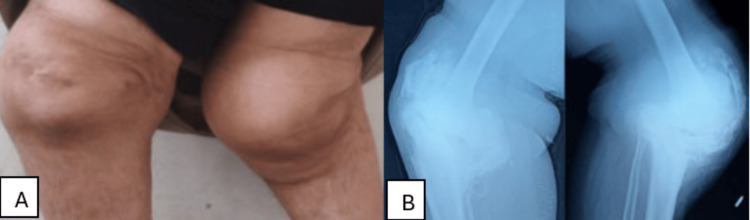
Imaging of Patient 3. (A) Clinical photograph of the knees demonstrating knee deformity. (B) Anteroposterior radiograph of both knees revealing bilateral destruction and malalignment.

On inspection, the severity of the knee deformities and axial malalignment were noted, which would compromise joint stability, particularly as this is a weight-bearing joint (Figure 4A). On physical examination of the knees, inspection revealed a varus deformity of the right knee and a valgus deformity of the left knee. There was also marked bilateral knee swelling that was non-tender to palpation and painless on passive range of motion. Coarse bony crepitus and a sensation of intra-articular loose bodies were noted during passive mobilization. Neurological examination revealed a positive Romberg sign and loss of deep sensation with absent patellar and Achilles deep tendon reflexes. Plain radiographs demonstrated rotational femorotibial subluxation, intra-articular bony fragments, and exuberant osteophytosis (Figure 4B).

Laboratory investigations in our male patient revealed an inflammatory syndrome and positive syphilis serology in serum, synovial fluid, and cerebrospinal fluid (Table 3).

**Table 3:** Syphilis serology results and normal ranges (Case 3). ESR: erythrocyte sedimentation rate; CRP: C-reactive protein; CSF: cerebrospinal fluid; TPHA: treponema pallidum hemagglutination; VDRL: venereal disease research laboratory.

	Results	Reference ranges
ESR	42	<20 mm
CRP	22	<5g mg/L
Serum syphilis serology	TPHA = 1:1280 (Reactive) VDRL = 1: 32 (Reactive)	Non-reactive
Synovial fluid syphilis serology	TPHA = 1:320 (Reactive) VDRL = 1:160 (Reactive)	Non-reactive
Cerebrospinal fluid syphilis serology	TPHA = 1:320 (Reactive) VDRL = 1:4 (Reactive)	Non-reactive

The cardiac evaluation was unremarkable. Following consultation with the infectious disease team, and due to the unavailability of aqueous crystalline penicillin G, our third patient also received a 14-day course of intravenous ceftriaxone (2 g daily). He was discharged with a scheduled six-month follow-up appointment for repeat VDRL testing, which subsequently returned negative.

In summary, we report three cases of tabetic arthropathy with distinct clinical presentations. The first case presented as destructive bilateral knee involvement associated with cardiac and neurological manifestations of syphilis. The second case was characterized by knee monoarthritis, also with concomitant cardiovascular and neurosyphilitic involvement. The third case demonstrated severe bilateral knee deformities with marked axial malalignment and joint instability, but without cardiac abnormalities. All three patients shared common neurological features: painless joint destruction, a positive Romberg sign, absent deep tendon reflexes, and loss of proprioception. Laboratory investigations confirmed active neurosyphilis in each case, with positive treponemal and non-treponemal tests in serum, cerebrospinal fluid, and synovial fluid. All patients were treated with intravenous ceftriaxone due to the unavailability of aqueous crystalline penicillin G, with an adequate serologic response at six-month follow-up in the two patients who completed monitoring.

## Discussion

Tabetic arthropathy is a destructive neurogenic joint disorder, classified among nervous osteoarthropathies due to the loss of deep pain and proprioceptive sensation [2]. Tabetic arthropathy represents a manifestation of tertiary syphilis, a sexually transmitted infection caused by the spirochete Treponema pallidum. This stage is highly contagious and fails to induce protective immunity, allowing persistent infection and late systemic complications [4]. Thanks to the implementation of early diagnosis and timely treatment of syphilis, neurosyphilis has become a rare complication. Nonetheless, the true incidence of the disease in Morocco remains undetermined [1]. Our series highlights the clinical heterogeneity of tabetic arthropathy. The bilateral knee involvement observed in two of our patients contrasts with most published cases, which predominantly report monoarticular disease, particularly of the knee or hip [1,2]. 

In our series, all three patients presented with the classical features of tabetic arthropathy, highlighting that, despite its rarity, the condition still occurs and can be identified through careful clinical and radiological evaluation. Its pathophysiology can be explained by two main theories. The neurotraumatic theory postulates that, in the absence of adequate protective sensory feedback, repeated mechanical microtraumas lead to progressive joint destruction. The neurovascular theory, on the other hand, emphasizes local vascular alterations as the primary mechanisms contributing to the joint degradation observed in neuropathic arthropathy [5]. In the patients presented, the combination of painless, progressive joint involvement and characteristic radiographic findings supports these underlying mechanisms.

Clinically, there is a pronounced dissociation between the severity of joint deformity and the absence or minimal perception of pain. Patients present with grossly deformed, swollen, yet painless joints. Sensory deficits predominantly include loss of deep sensation and proprioception, as observed in our three patients. In advanced stages, patients may exhibit ataxia and a positive Romberg’s sign. Osteotendinous reflexes are abolished in approximately 90% of individuals with tabes dorsalis. Motor dysfunction is characterized by an imbalance between extensor and flexor muscle groups, resulting in progressive joint deformity and ligamentous laxity, which predisposes to joint subluxation. Vegetative disturbances may include cutaneous dryness, localized hyperemia, and edema [2].

The radiographic features of a Charcot joint are classically characterized by the six “D”s: Density changes, Destruction, Debris, Distension, Disorganization, and Dislocation [4]. Additional manifestations may include tenosynovitis, periostitis, myositis, and myonecrosis [6].

The association with cardiovascular syphilis in our first two cases is also noteworthy. While aortic regurgitation secondary to syphilitic aortitis is well described, its coexistence with destructive arthropathy in the same patient remains rarely documented in the modern antibiotic era. To our knowledge, few contemporary cases report concomitant cardiovascular and articular manifestations of tertiary syphilis [7,8].

However, other cardiovascular events may also occur, such as heart failure, atrial fibrillation, ischemic stroke, hemorrhagic stroke, and venous thromboembolism. There is also a significantly increased risk of all-cause mortality associated with these conditions [9]. 

This multisystem involvement underscores the importance of systematic cardiac and neurological evaluation in patients presenting with painless destructive arthropathy.

As with other forms of syphilis, treatment involves penicillin G, which is administered to prevent the progression and development of late-stage syphilitic lesions. In cases of penicillin allergy or unavailability, ceftriaxone is a viable alternative, administered at a dose of 1-2 g per day for 10-15 days [10].

The serologic response to ceftriaxone observed in our first and third patients at six months aligns with CDC guidelines supporting ceftriaxone 1-2 g IV daily for 10-14 days as an effective alternative when aqueous crystalline penicillin G is unavailable [11].

Orthopedic management is considered on a case-by-case basis but remains generally unsatisfactory. The indications for prosthetic arthroplasty are limited due to the extent of joint destruction, local tissue condition, and the elevated risk of prosthetic loosening [2]. The cases we report illustrate these limitations, emphasizing the challenges in achieving satisfactory functional outcomes even with appropriate medical and surgical interventions.

## Conclusions

Tabetic arthropathy, though rare, persists in the antibiotic era and presents diagnostic and therapeutic challenges. This series illustrates its heterogeneous presentation, including concomitant cardiovascular syphilis, and highlights the importance of early suspicion in painless destructive arthropathy with neurological signs. Ceftriaxone represents a viable alternative to penicillin G when it is unavailable. Given the global resurgence of syphilis, clinicians must remain vigilant to prevent progression to irreversible tertiary complications through timely diagnosis and treatment.
